# Estimation of the Orexin Receptor Occupancy From Human Plasma Pharmacokinetics of Vornorexant, a Novel Dual Orexin 1/2 Receptor Antagonist for the Treatment of Insomnia

**DOI:** 10.1002/prp2.70217

**Published:** 2026-02-01

**Authors:** Shunsuke Kamigaso, Yoshihiro Konno, Hirohiko Hikichi, Daiji Kambe, Yoko Mano, Yuichi Tokumaru, Haruyuki Mori, Hironori Yamasaki, Yukihiro Chino, Kenji Hachiuma, Akiko Mizuno‐Yasuhira

**Affiliations:** ^1^ Research Center Taisho Pharmaceutical Co., Ltd. Saitama Japan; ^2^ Development Headquarters Taisho Pharmaceutical Co., Ltd. Toshima Japan

**Keywords:** orexin, pharmacokinetic, receptor occupancy, TS‐142, vornorexant

## Abstract

Vornorexant is a novel dual orexin receptor antagonist (DORA) for the treatment of insomnia with a short elimination half‐life. Estimation of the human orexin receptor occupancy can provide insight into the duration of the sleep‐promoting effect of DORAs. Herein, we developed a pharmacokinetic‐receptor occupancy (PK/RO) model for OX_1_ and OX_2_ receptors in rats and estimated their receptor occupancy in humans based on the human plasma concentrations after oral administration of vornorexant. The estimated occupancy of the human OX_2_ receptor, which is primarily involved in sleep induction, exceeded 70% at 30 min after dosing of therapeutic doses of 5 and 10 mg. The human OX_2_ receptor occupancy at 6 h post‐dose of 5 and 10 mg was estimated to be approximately 50% and 60%, respectively, comparable to the occupancy necessary to exert sleep‐promoting effects in rats. Occupancy of the human OX_1_ receptor by vornorexant was higher than that of the OX_2_ receptor. The estimated human OX_1_ and OX_2_ receptor occupancy decreased with short half‐lives depending on the plasma concentration. These results suggest that vornorexant exerts rapid sleep‐promoting effects and maintains sleep for at least 6 h, and these estimations of the OX_2_ receptor occupancy could support the clinical results. Vornorexant may have a favorable PK/RO profile for rapid sleep onset and sufficient sleep maintenance with minimal next‐day residual effects in humans.

**Abbreviations**
2‐SORA: selective orexin 2 receptor antagonist
AUC_0‐∞_
: area under the concentration‐time curve from time 0 to infinity
BMI: body mass index
CI: confidence interval
CL/F: apparent clearance
*C*_max_
: maximal concentration
CNS: central nervous system
CSF: cerebrospinal fluid
CV: coefficient of variation
DORA: dual orexin receptor antagonist
EC_50_
: 50% effective concentration
*E*_max_
: maximal effect
FDA: U.S. Food and Drug Administration
fu: unbound plasma fraction
HPLC: high‐performance liquid chromatography
*k*_a_
: absorption rate constant
LC–MS/MS: liquid chromatography–tandem mass spectrometry
ORA: orexin receptor antagonist
OX_1_ receptor: orexin‐1 receptor
OX_2_ receptor: orexin‐2 receptor
PD: pharmacodynamics
PK: pharmacokinetic
SD: Standard Deviation
*t*_1/2_
: half‐life
*t*_max_
: time to peak concentration
Vd/F: apparent volume of distribution

## 1 Introduction

Insomnia is characterized by difficulties in sleep onset and/or sleep maintenance. Benzodiazepine‐like compounds, including Z‐drugs (e.g., zolpidem), are widely prescribed for insomnia and have improved pharmacotherapy for insomnia [1], but their long‐term use is associated with an increased risk of falls and driving accidents, drug abuse, and next‐day cognitive impairment [2]. As safer alternatives, orexin receptor antagonists have been developed as hypnotics with different mechanisms of action from those of benzodiazepine‐like compounds. Orexins, which were discovered in 1998, comprise two neuropeptides, orexin‐A and orexin‐B [3, 4]. Orexins activate their associated G protein‐coupled receptors, the orexin‐1 (OX_1_) receptor and orexin‐2 (OX_2_) receptor, and regulate the sleep–wake cycle through its arousal‐promoting effect [5, 6, 7]. Orexin deficiency has also been reported to be associated with sleep–wake disorders such as narcolepsy type 1 symptoms [8, 9]. The blockade of OX_2_ receptor plays a primary role in sleep regulation, and the blockade of OX_1_ receptor is known to act synergistically with OX_2_ receptor to regulate sleep [10]. Dual orexin receptor antagonists (DORAs) such as suvorexant, lemborexant, and daridorexant are approved for the treatment of insomnia and DORAs have been shown to be not only effective but also well tolerated [11, 12, 13]. However, the long elimination half‐life (*t*_1/2_) of DORAs may be associated with an increased risk of next‐day residual effects, such as impairment of cognitive function and driving performance [14, 15]. Therefore, we developed a potent DORA with favorable PK properties, including rapid absorption and a relatively short *t*_1/2_ [16]. In a phase I trial, vornorexant was found to be rapidly absorbed and to have a shorter half‐life than other DORAs (1.32–3.25 h for vornorexant [17], approximately 12 h for suvorexant [11], 17–19 h of effective half‐life for lemborexant [12], and approximately 8 h for daridorexant [13]). A phase III trial demonstrated that vornorexant significantly improved subjective sleep latency and sleep maintenance and was well tolerated in patients with insomnia [18]. At the present, vornorexant is approved for the treatment of insomnia in Japan.

The sleep‐promoting effect depends on not only the pharmacokinetics but also the binding potency and kinetics of the drugs to the orexin receptors. It is a useful approach for evaluating drug profile to estimate the duration of the sleep‐promoting effect from the relationship between the exposure and receptor occupancy [19, 20]. The human OX_2_ receptor occupancy for suvorexant has been estimated based on the effective occupancy obtained from animal experiments for the sleep‐promoting effect, and the next‐day carryover risk has also been evaluated [19]. Previous findings have shown that DORAs such as suvorexant and daridorexant exert their sleep‐promoting effects through high OX_2_ receptor occupancy of over 65%, while selective orexin 2 receptor antagonists (SORAs) such as seltorexant and MK‐1064 were also found to show high OX_2_ receptor occupancy of about 50% and 90%, respectively [21, 22]. From these findings, orexin receptor antagonists are considered to exert their sleep‐promoting effects through high OX_2_ receptor occupancy of at least 50% or more, although the occupancy threshold may vary depending on the mode of action and the compound.

The sleep‐promoting effects and OX receptor occupancy of vornorexant have been evaluated in rats, and vornorexant has been shown to significantly decrease the sleep‐onset latency and increase the total sleep duration at doses of more than 1 mg/kg, with OX_1_ and OX_2_ receptor occupancy of approximately 50% or more [23]. However, the human OX_1_ and OX_2_ receptor occupancy of vornorexant at clinical doses have not yet been investigated, and the receptor occupancy to induce sleep at the onset and maintain sleep throughout the night remains unclear in humans.

Herein, we developed a PK/RO model for OX_1_ and OX_2_ receptors in rats and estimated the human receptor occupancy of OX_1_ and OX_2_ based on the plasma concentrations after oral administration of vornorexant at clinical doses. Furthermore, the sleep‐promoting effects of vornorexant in clinical trials are discussed based on the estimated occupancy.

## 2 Materials and Methods

### 2.1 Materials

Vornorexant (Mw: 447.46) was synthesized at Taisho Pharmaceutical Co. Ltd. (Saitama, Japan).

### 2.2 Animals

Male Wistar rats (10–12 weeks old, Charles River, Yokohama, Japan) were used to investigate receptor occupancy, conduct electroencephalographic recordings, and estimate pharmacokinetics. The animals were maintained under a 12‐h light/dark cycle in a temperature‐ and humidity‐controlled room, with food and water made available ad libitum. All animal study protocols were approved by the Animal Care and Use Committee in Taisho Pharmaceutical Co. Ltd.

### 2.3 Effects of Vornorexant on the Sleep–Wake States in Rats

The effects of vornorexant on the sleep–wake states were investigated in Wistar rats (10 rats/group) surgically implanted with electrodes for electroencephalogram (EEG) and electromyogram (EMG) recordings, as described in our previous report [16]. The rats were individually transferred to an acrylic chamber within an electrically shielded sound‐proof box (light on: 5:00–17:00 h) to acclimate to the measurement environment. Vornorexant was orally administered to rats at doses of 1, 3, and 10 mg/kg in the previous study [16], and at the dose of 0.3 mg/kg in the present study, within 10 min prior to the onset of the dark period. EEG and EMG recordings were conducted for 2 h after the administration. The sleep–wake states were classified every 20 s as wakefulness or sleep based on the EEG and EMG records.

### 2.4 Sleep Efficacy‐Orexin Receptor Occupancy Relationship in Rats

The sleep‐inducing efficacy of vornorexant was evaluated in rats at doses of 1, 3, and 10 mg/kg in the previous study [16], and at 0.3 mg/kg in the present study. The mean of percent changes in total sleep duration during a 2‐h period in the vornorexant‐treated groups as compared with the vehicle‐treated group was calculated. The OX_1_ and OX_2_ receptor occupancy of vornorexant at doses of 0.3, 1, 3, and 10 mg/kg in rats was evaluated at 0.5 h post‐dose in the previous study [23].

### 2.5 Pharmacokinetics‐Orexin Receptor Occupancy (PK/RO) Relationship in Rats

A modeling approach was implemented for determining the PK/RO relationship, in which the plasma concentrations of vornorexant and the OX_1_ and OX_2_ receptor occupancy at 0.5, 1, 2, 4, and 6 h post administration of vornorexant at 3 mg/kg were evaluated in the rats in the previous study [23]. The Sigmoid *E*_max_ model (Phoenix WinNonlin version 8.5, Certara, Princeton, NJ) was used for the analysis of the PK/RO relationship.

$$\text{Sigmoid}\;E_{\max}\;\text{model}:\mathrm{RO}\;\left(\%\right)=\frac{E_{\max}\times C_{u}^{\gamma }}{{\mathrm{EC}}_{50}^{\gamma }+C_{u}^{\gamma }}$$

where *E*_max_ was fixed at 100 for this analysis, EC_50_ is the concentration at half‐maximal receptor occupancy, *γ* is the hill coefficient, and *C*_u_ is the unbound plasma concentration of vornorexant. The unbound plasma concentration was calculated for the PK/RO analysis based on the plasma protein binding rates of 92.6% in rats and 94.5% in humans.

### 2.6 Clinical Pharmacokinetic Study

The pharmacokinetic data from two clinical pharmacology trials conducted to evaluate the effects of vornorexant on the QT/QTc interval (Study 209: NCT04873323) and the effects of food on the pharmacokinetics of vornorexant (Study 304: NCT05707897) were used in this analysis. Both studies were carried out by Taisho Pharmaceutical Co. Ltd. in accordance with the Declaration of Helsinki and with the approval of the relevant local ethics committees. Healthy Japanese subjects were enrolled in these studies, and the demographic characteristics are shown in Table 1. In Study 209, each subject under the fasting condition received either a single dose of 10 mg or 30 mg of vornorexant tablets, 400 mg of moxifloxacin, and placebo once during each of 4 treatment periods in a balanced order. In Study 304, each subject received a single oral dose of 10 mg of vornorexant in 2 treatment periods (under the fasting and fed conditions). All the treatments were undertaken under the admitted conditions of a phase I trial, with a washout period of at least 7 days between the conditions. Blood samples were collected from 55 subjects (Study 209) and 12 subjects (Study 304), and the pharmacokinetic data determined for a vornorexant dose of 10 mg administered under the fasting condition were included in the analyses in the present study.

**TABLE 1:** Demographic data of the patients in Study 209 and Study 304.

	Dose (mg)	*N*	Age (year)		Weight (kg)		BMI	
			Mean (SD)	Min, max	Mean (SD)	Min, max	Mean (SD)	Min, max
Study 209	10	55	25.7 (4.9)	20, 39	56.21 (7.77)	41.5, 73.9	21.16 (1.83)	17.8, 24.3
Study 304	10	12	24.9 (4.7)	20, 35	62.79 (5.05)	52.3, 67.5	21.15 (1.45)	18.7, 23.3

Abbreviation: BMI, body mass index.

### 2.7 Human Pharmacokinetic Analysis

Blood samples were collected in containers containing EDTA‐2K as an anticoagulant at the following time‐points: pre‐dose, 0.5, 1, 2, 3, 4, 6, 8, 12, 24, and 48 h post‐dose in Study 209; and pre‐dose, 0.25, 0.5, 0.75, 1, 1.5, 2, 3, 4, 5, 6, 9, 12, 18, and 24 h post‐dose in Study 304. Plasma samples were stored at −70°C or below until analysis. The human plasma concentrations of vornorexant were determined using validated LC–MS/MS assays as described in the previous report [17]. The plasma concentrations of vornorexant were analyzed only under the fasting condition, which closely reflects actual clinical practice, as follows:

The PK parameters of vornorexant were determined from the plasma concentrations by non‐compartmental analysis using Phoenix WinNonlin (version 8.5). The PK parameters calculated were the *C*_max_, time to maximum concentration (*t*_max_), area under the plasma concentration‐time curve from time 0 to the time of the last quantifiable concentration (AUC_0–last_), AUC from time 0 to infinity (AUC_0–inf_), and apparent plasma terminal elimination half‐life (*t*_1/2_).

### 2.8 Simulation of the Pharmacokinetics in Humans

A one‐compartment model with first‐order absorption was used to fit the observed plasma concentrations of vornorexant in each of the 67 subjects (55 subjects in Study 209, 12 subjects in Study 304), considering inter‐individual pharmacokinetic variability, using Phoenix WinNonlin (version 8.5). The plasma concentrations of vornorexant after the oral administration of 10 mg in each of the 67 subjects were individually simulated using the corresponding estimated PK parameters: absorption rate constant (*k*_a_), apparent clearance (CL/F), and apparent volume of distribution (Vd/F). Since the dose proportionality of the pharmacokinetics of vornorexant in the dose range of 1 to 10 mg was confirmed [17], the plasma concentrations of vornorexant at 5 mg were simulated based on the dose linearity from the concentrations of vornorexant at 10 mg.

### 2.9 Estimation of the OX_1_ and OX_2_ Receptor Occupancy in Humans

We hypothesized that the relationship between the unbound plasma concentrations and receptor occupancy would be valid in humans as in rats. The above PK/RO model developed based on this relationship in rats was extrapolated to humans. The simulated human plasma concentrations at clinical doses of 5 and 10 mg and the protein binding rates were used to calculate the unbound plasma concentrations for each of the 67 subjects. Subsequently, the human receptor occupancy at these unbound plasma concentrations was individually estimated using the PK/RO model (Phoenix WinNonlin version 8.5). The half‐lives of the OX_1_ and OX_2_ receptor occupancy from 6 to 12 h post‐dose were calculated using Phoenix WinNonlin (version 8.5).

## 3 Results

### 3.1 Sleep‐Promoting Effects and OX_1_ and OX_2_ Receptor Occupancy in Rats

**FIGURE 1:**
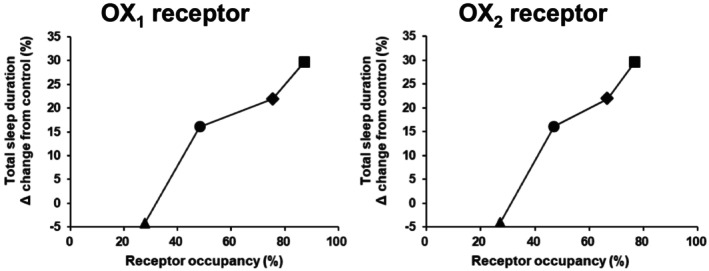
Relationship of sleep efficacy and OX_1/2_ receptors occupancy by vornorexant in rats. The sleep efficacy in rats was evaluated in a previous study following administration of vornorexant at doses of 1, 3, and 10 mg/kg [16] and in the present study following administration at the dose of 0.3 mg/kg. The OX_1/2_ receptor occupancy in rats was evaluated following administration of vornorexant at the doses of 0.3, 1, 3, and 10 mg/kg in the previous study [23]. Total sleep duration for 2 h was evaluated by polysomnography recordings and the OX_1/2_ receptor occupancy at 0.5 h post‐dose (around *t*_max_) was evaluated after a single oral administration of vornorexant to rats at doses of 0.3 (▲), 1 (●), 3 (◆), and 10 (■) mg/kg. The mean percent change in the total sleep duration in the vornorexant‐treated groups as compared with the vehicle‐treated group (control) (*n* = 10) versus the mean percent OX_1/2_ receptor occupancy (*n* = 5) is represented. Vornorexant significantly increased the total sleep duration at doses of 1 mg/kg or higher.

The effect of vornorexant on the sleep–wake states was evaluated for 2 h after oral administration just before the start of the dark period in rats using electroencephalogram and electromyogram recording in our previous study [16]. Vornorexant was shown to significantly increase the total sleep time in a dose‐dependent manner from 1 mg/kg, but did not increase at 0.3 mg/kg. The OX_1_ and OX_2_ receptor occupancy by vornorexant were also investigated using ex vivo binding methods in rats [23]. At 0.5 h (around *t*_max_) following the minimum effective dose (1 mg/kg) of vornorexant, the OX_1_ and OX_2_ receptor occupancy of vornorexant were both approximately 50%, and the occupancy increased in a dose‐dependent manner. At the dose of 0.3 mg/kg (non‐effective dose), on the other hand, the occupancy rates of both receptors were approximately 25%. The relationship between the changes in the total sleep time relative to the vehicle‐treated groups and the OX_1_ and OX_2_ receptor occupancy in rats at doses of 0.3, 1, 3, and 10 mg/kg of vornorexant are shown in Figure 1.

### 3.2 Plasma Concentrations and OX_1_ and OX_2_ Receptor Occupancy (PK/RO) in Rats

**FIGURE 2:**
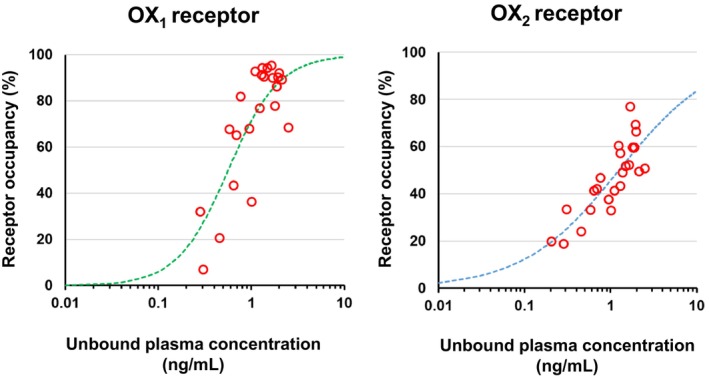
Unbound plasma concentration of vornorexant versus the OX_1_ or OX_2_ receptor occupancy by vornorexant in rats. A PK/RO model analysis was applied to the data evaluated in our previous report [23]. The unbound plasma concentration of vornorexant and the OX_1/2_ receptor occupancy were evaluated at 0.5, 1, 2, 4, and 6 h (each point: *n* = 4 or 5) after the administration of 3 mg/kg of vornorexant to rats. The relationship of the OX_1/2_ receptor occupancy and the unbound plasma concentration of vornorexant determined using the sigmoid *E*_max_ model is represented by the dashed line.

The PK/RO relationship between the unbound plasma concentrations and OX_1_ or OX_2_ receptor occupancy by vornorexant in rats at 0.5, 1, 2, 4, and 6 h after administration of vornorexant at 3 mg/kg is shown in Figure 2. For both OX_1_ and OX_2_ receptors, the sigmoid *E*_max_ model provided a good fit to the unbound plasma concentration‐receptor occupancy relationship. The calculated parameters are shown in Table 2. The EC_50_ for the OX_1_ receptor was 0.563 ng/mL (CV: 10.6%) and that for the OX_2_ receptor was 1.25 ng/mL (CV: 10.1%). The fitted line of PK/RO relationship well captured the observed values, and it was considered possible to estimate the OX_1_ and OX_2_ receptor occupancy by vornorexant from the unbound plasma concentrations in the subsequent analyses.

**TABLE 2:** Modeling parameters of the PK/RO relationship in rats.

Parameter	OX_1_ receptor		OX_2_ receptor	
	Value	CV (%)	Value	CV (%)
EC_50_ (ng/mL)	0.563	10.6	1.25	10.1
Hill coefficient	1.59	17.4	0.779	10.8
*E*_max_ (%)	100	—	100	—

*Note:* Sigmoid *E*_max_ model was applied to the PK/RO relationship in rats. Values expressed are the means with CV.

### 3.3 Human Pharmacokinetics

**FIGURE 3:**
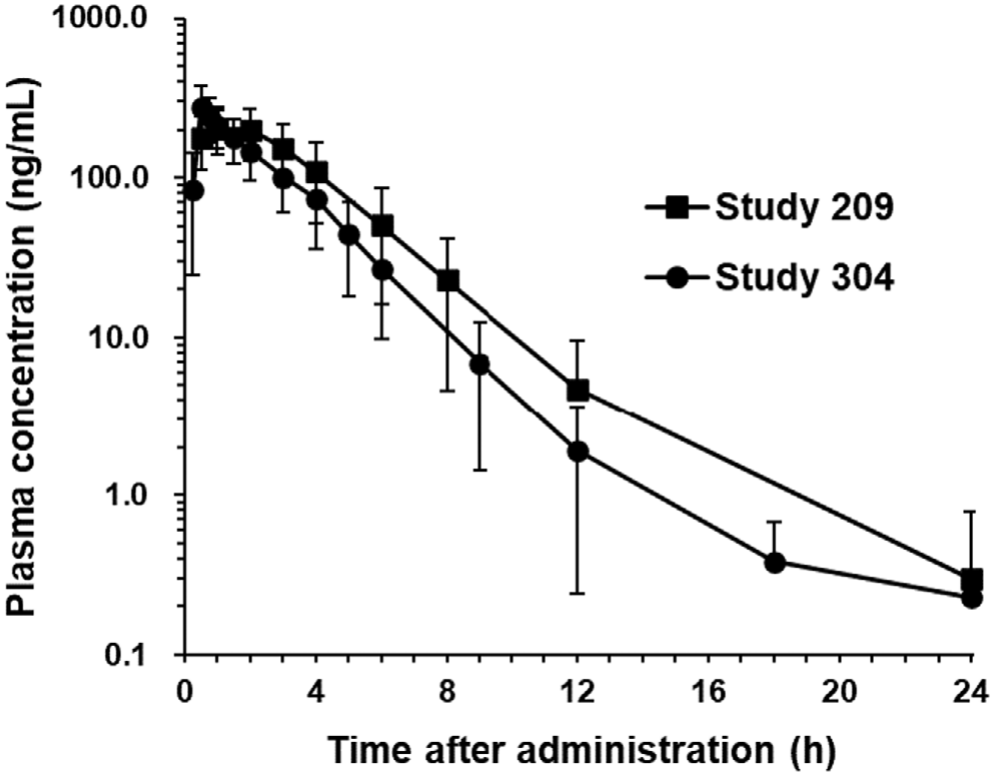
Plasma concentration‐time profiles of vornorexant following single administration of 10 mg of vornorexant to healthy subjects (Study 209 and Study 304). The concentrations are expressed in a semi‐logarithmic scale. Graph shows the arithmetic means with SD. *n* = 55 for Study 209, *n* = 12 for Study 304.

The observed plasma concentration‐time profiles and the PK parameters of vornorexant (10 mg) in Study 209 and Study 304 are shown in Figure 3 and Table 3, respectively. Similar plasma concentration‐time profiles were observed in both Studies. The plasma concentration of vornorexant reached a *C*_max_ at 0.50–1.18 h post‐dose, and then decreased with a *t*_1/2_ ranging from 2.13 to 2.29 h in each Study (Figure 3, Table 3).

**TABLE 3:** Plasma pharmacokinetic parameters following administration of vornorexant at a single oral dose under the fasting condition in healthy subjects (Study 209 and Study 304).

	Dose (mg)	*N*		*C*_max_ (ng/mL)	*t*_max_ (h)	AUC_0–last_ (h × ng/mL)	AUC_0–inf_ (h × ng/mL)	*t*_1/2_ (h)
Study 209	10	55	Observed	225 (66.0)	1.18 (0.683, 3.18)	943 (390)	946 (390)	2.29 (2.96)
Study 304	10	12	Observed	279 (9.67)	0.500 (0.500, 3.00)	720 (237)	720 (237)	2.13 (0.185)
Total	10	67	Observed	235 (74.6)	1.18 (0.500, 3.18)	903 (376)	906 (376)	2.26 (2.68)
			Simulated	208 (63.2)	1.20 (0.200, 2.20)	916 (373)	916 (373)	1.97 (0.855)

*Note:* Data shown are the arithmetic means (SD), except for *t*_max_, for which the median value is shown (minimum, maximum).

**FIGURE 4:**
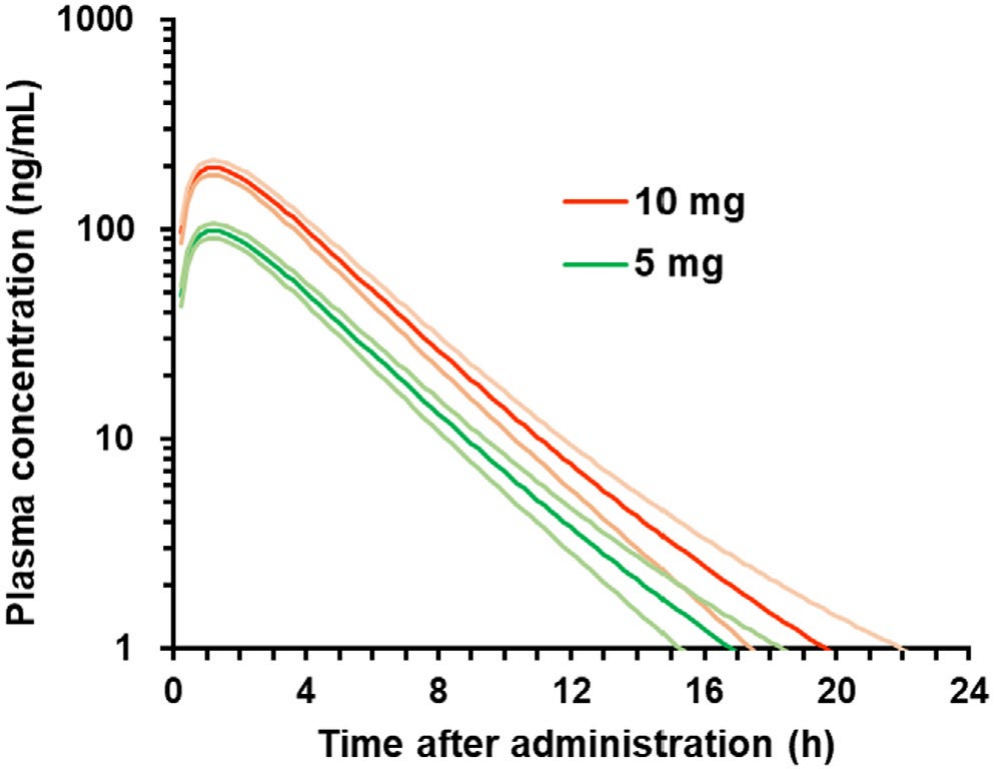
Simulated plasma concentration‐time profiles of vornorexant after single oral administration of 5 mg (green line) or 10 mg (orange line) of vornorexant to each of the healthy subjects (Study 209 and Study 304, total *n* = 67). The values shown are means (bold line) with 2‐sided 95% confidence intervals (thin line).

The observed plasma concentrations of vornorexant administered at the dose of 10 mg in each subject in Study 209 and Study 304 were individually analyzed using a one‐compartment model with first‐order absorption, and the PK parameters of the one‐compartment model in each subject were calculated for simulation of the PK and RO in humans. The plasma concentrations of vornorexant after oral administration at the doses of 5 and 10 mg were individually simulated using the corresponding PK parameters calculated in each subject (Figure 4). The PK parameters of the simulated plasma concentrations at the dose of 10 mg were comparable levels to those of the observed plasma concentrations (Table 3, observed vs. simulated in total).

### 3.4 Estimation of OX_1_ and OX_2_ Receptor Occupancy in Humans

**FIGURE 5:**
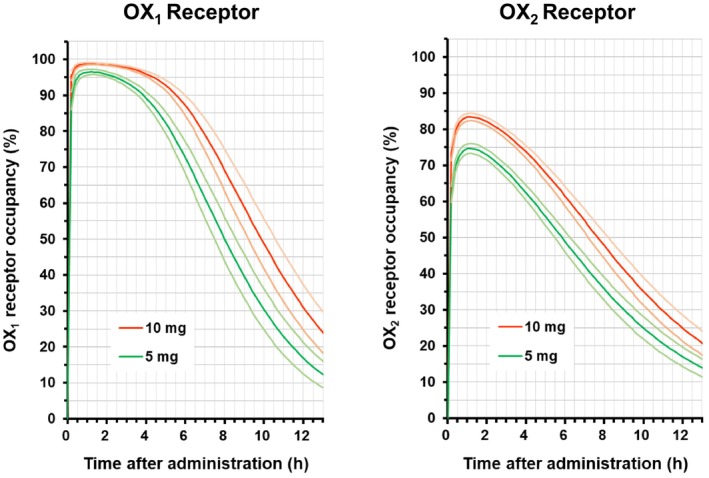
Estimated OX_1_ and OX_2_ receptor occupancy‐time profiles of vornorexant in humans. The parameters describing the rat PK/RO relationship shown in Table 2 and the human PK parameters in each of the subjects (*n* = 67) shown in Table 4 were used for estimation of the OX_1_ and OX_2_ receptor occupancy in humans by vornorexant. Means (bold line) with 2‐sided 95% confidence intervals (thin line) are expressed for the 5 mg (green line) and 10 mg (orange line) doses.

The human OX_1_ and OX_2_ receptor occupancy were estimated based on the estimated PK/RO relationship in rats and the simulated unbound human plasma concentrations in each subject. The modeling parameters of the PK/RO relationship and the human PK are shown in Tables 2 and 4, respectively. The human OX_1_ and OX_2_ receptor occupancy estimated in the present study are shown in Figure 5. The human OX_1_ and OX_2_ receptor occupancy at 0.5 h post‐dose of 5 and 10 mg were estimated to be approximately 95% and 98% for the OX_1_ receptor, and approximately 70% and 80% for the OX_2_ receptor, respectively. The estimated OX_1_ and OX_2_ receptor occupancy by vornorexant reached almost maximum rates at approximately 1.0 h post‐dose, and the OX_1_ and OX_2_ receptor occupancy at 6 h post‐dose of 5 and 10 mg were estimated to be approximately 70% and 85% for the OX_1_ receptor, and approximately 50% and 60% for the OX_2_ receptor, respectively. After 6 h, the OX_1_ and OX_2_ receptor occupancy at 5 and 10 mg decreased with *t*_1/2_ values of 2.83 and 3.99 h for the OX_1_ receptor, and 3.91 and 4.58 h for the OX_2_ receptor, respectively, comparable to the observed *t*_1/2_ (2.26 ± 2.68 h) in the plasma concentration. The OX_1_ and OX_2_ receptor occupancy decreased to less than 1.3% and 2.7%, respectively, at 24 h after the administration.

**TABLE 4:** PK parameters applied to estimation of the OX_1_ and OX_2_ receptor occupancy in humans.

Parameter	Point estimate	(95% CI)
*k*_a_ (h^−1^)	3.57	(2.01, 5.13)
CL/F (L/h)	13.1	(11.4, 14.7)
Vd/F (L)	34.3	(30.2, 38.3)

*Note:* The parameters were individually calculated from the observed plasma concentrations in each of the 67 subjects in Study 209 and Study 304 by using a one‐compartment model with first‐order absorption. The values are expressed as point estimates with 95% CI.

## 4 Discussion

Herein, we developed a PK/RO model for determining the OX_1_ and OX_2_ receptor occupancy by vornorexant in rats and estimated the OX_1_ and OX_2_ receptor occupancy in humans from the human PK to evaluate the clinical PK/PD profile of vornorexant. We demonstrated that vornorexant is expected to exert rapid sleep‐promoting effects and maintain sleep throughout the night based on the estimated human receptor occupancy.

Vornorexant has comparable affinity and antagonistic activity to the OX receptor in rats and humans, with no species differences in the pharmacological profile of vornorexant [16, 23]. Vornorexant also has high membrane permeability, and the concentration of vornorexant in the CSF is comparable to the unbound concentration in the plasma and changes with the same time profiles as the plasma concentrations in rats after oral administration [24], suggesting that the distribution of vornorexant to the CNS is not restricted by transporters. It is considered that the CNS distribution via passive diffusion does not differ among species. Furthermore, the OX_1_ and OX_2_ receptor occupancy by vornorexant in the rat brain changes depending on its plasma concentration–time profile, suggesting that the pharmacodynamic effects of vornorexant may be driven by its pharmacokinetics [23]. Based on these findings, we considered that the human receptor occupancy by vornorexant can be estimated from the unbound plasma concentration in humans using the rat PK/RO relationship, leading to verification of the sleep‐promoting effects in humans.

Vornorexant exerted significant sleep‐promoting effects at doses of 1 mg/kg or more in rats [23]. The OX_2_ receptor is primarily involved in sleep regulation [10]. At the minimum effective dose of 1 mg/kg, the OX_2_ receptor occupancy by vornorexant in rats was approximately 50%. In previous reports, the sleep‐promoting effect in humans has been predicted based on the effective occupancy for OX_2_ receptor obtained from animal studies [19, 21]. Similarly, we hypothesized that vornorexant exerts a sleep‐promoting effect in humans when the estimated human OX_2_ receptor occupancy by vornorexant exceeds the effective occupancy level (approximately 50%) obtained from rat study, and we investigated its relevance to the clinical efficacy.

Rapid drug absorption leads to a high OX_2_ receptor occupancy and a sleep‐promoting effect. Vornorexant is designed to reduce lipophilicity while having a high membrane permeability for rapid absorption [16]. In fact, vornorexant was rapidly absorbed in healthy subjects after oral administration under the fasting condition at the clinical high dose of 10 mg, with a *t*_max_ of 0.50 h in Study 304 and of 1.18 h in Study 209. According to the label information, the *t*_max_ of vornorexant is comparable or relatively short as compared with that of other DORAs (2 h for suvorexant, 1–3 h for lemborexant, and 1–2 h for daridorexant) after single administration under the fasting condition [11, 12, 13]. Because of the rapid increase in the plasma concentration of vornorexant after administration, the human OX_2_ receptor occupancy by vornorexant at 0.5 h post‐dose was estimated to be high values of more than 70%, exceeding the effective occupancy of approximately 50% in rats (Figure 5), indicating that vornorexant can sufficiently occupy OX_2_ receptor around 0.5 h post‐dose when administered at clinical doses (5 mg, 10 mg). In a phase IIa clinical trial assessed by polysomnography (PSG) in the dose range of 5–30 mg, vornorexant significantly shortened the latency to persistent sleep measured by PSG as compared with placebo [25]. In addition, the sleep‐promoting effect of vornorexant was also confirmed in a phase III randomized placebo‐controlled clinical trial, in which the subjective sleep latencies assessed by sleep diary were significantly shortened following oral dosing with both 5 and 10 mg of vornorexant as compared with placebo [18]. In the present study, vornorexant was estimated to show sufficient occupancy of the OX_2_ receptor within 30 min after administration in humans, lending support to the rapid sleep latency demonstrated in these clinical trials.

Vornorexant (5 to 30 mg) significantly shortened the wake after sleep onset as compared with placebo in the phase IIa clinical trial assessed by PSG [25]. Furthermore, in the phase III randomized controlled clinical trial, vornorexant (5 mg, 10 mg) has been shown to exert sleep‐maintenance effect as assessed by subjective sleep efficiency (sSE), as compared with placebo [18]. In the present study, the OX_2_ receptor occupancy by vornorexant at 6 h post‐dose was estimated to be approximately 50% for 5 mg and 60% for 10 mg, being comparable or higher than the effective occupancy level (approximately 50%) in rats. These estimations suggest that vornorexant can exert sleep‐promoting effects for at least 6 h. The sleep‐maintenance effect predicted in this study based on the human OX_2_ receptor occupancy was considered as lending support to the objective and subjective clinical efficacy of vornorexant. Moreover, the unbound plasma concentration of vornorexant in humans at 6 h post‐dose was 3.15 nmol/L for 5 mg and higher than the IC_50_ value (1.76 nmol/L) [23] in function assay for human OX_2_ receptor, which also lends support to the notion that vornorexant can exert the sleep‐promoting effect for 6 h. In addition, the occupancy of the OX_2_ receptor by vornorexant decreased with a short half‐life (RO *t*_1/2_: 3.91 h for 5 mg, 4.58 h for 10 mg), similar to the plasma concentrations, suggesting that vornorexant may be associated with a low risk of next‐day residual effects.

Although there are no clear reports about the expected duration of action of hypnotic drugs, several meta‐analyses have indicated that the sleep duration is not only related to the quality of life, but also to the risk of all‐cause mortality, cardiovascular events, and cancer [26, 27, 28]. According to these meta‐analyses, 7 h of sleep is recommended to prevent premature death, and the aforementioned risks associated with 6 h of sleep were almost the same as those associated with 7 h of sleep, whereas more than 8 h of sleep increased the risk level [26, 27]. Therefore, hypnotic drugs such as vornorexant, which exert a sleep‐promoting effect for at least 6 h, may maintain sleep for the ideal duration.

The occupancy of the OX_1_ receptor by vornorexant was also estimated in this study. The estimated OX_1_ receptor occupancy at 6 h post‐dose was approximately 70% for 5 mg and 85% for 10 mg, values higher than the OX_2_ receptor occupancy at the same time (OX_2_ ROs: approximately 50% and 60%, respectively). However, several studies have suggested that blockade of the OX_1_ receptor may play a complementary role in sleep–wake regulation, and that blockade of the OX_2_ receptor has the primary role in the promotion of sleep [10, 29, 30]. The 1‐SORA JNJ‐54717793 had a minimal effect on spontaneous sleep in rats and mice [31], and blockade of the OX_1_ receptor attenuated OX_2_ receptor antagonism‐induced sleep promotion in rats [32]. Since the impact of OX_1_ receptor occupancy on the sleep‐promoting effect of DORAs remains controversial, we predicted the sleep‐promoting effects of vornorexant only from OX_2_ receptor occupancy, and not OX_1_ receptor occupancy. It is noteworthy that the blockade of both OX_1_ receptor and OX_2_ receptor may have different effects from the blockade of OX_2_ receptor alone [33, 34]. Therefore, the impact of OX_1_ receptor occupancy cannot be fully ruled out.

In the present study, we estimated the human OX_2_ receptor occupancy by vornorexant from a rat PK/RO model, under the assumption of the absence of any species differences in the CNS distribution or relationship of the unbound plasma drug concentration to OX_2_ receptor occupancy between rats and humans. However, the actual OX_2_ receptor occupancy is still not known. Further investigations using imaging techniques such as positron emission tomography are necessary for precise estimation of the OX_2_ receptor occupancy in humans. In addition, given the individual differences in human pharmacokinetics, some patients may exhibit higher receptor occupancy and longer half‐life than the average values described. It is, therefore, important to note that the disappearance time of the sleep‐promoting effect varies among individuals.

Herein, we estimated the human OX_1_ and OX_2_ receptor occupancy by vornorexant based on the PK/RO relationship in rats and the human PK. The present findings suggest that vornorexant shows sufficient occupancy of the OX_2_ receptor immediately after administration, lending support to its effect of inducing rapid sleep onset in humans. Our findings also suggested that vornorexant occupies the OX_2_ receptor to exert the sleep‐promoting effects for 6 h post‐dose. The estimated occupancy of the OX_1/2_ receptors decreased with a short half‐life, similar to the plasma concentrations. The sleep‐promoting effect of vornorexant, determined based on the human OX_2_ receptor occupancy estimated in this study, was consistent with the clinical results. Vornorexant is expected to become a useful hypnotic drug with a favorable PK/RO profile, inducing rapid sleep onset and showing sleep‐maintenance effect throughout the night with minimal next‐day residual effects.

## Author Contributions

Participated in research design: Yoshihiro Konno, Shunsuke Kamigaso, Hirohiko Hikichi, Yuichi Tokumaru, Yoko Mano, Yukihiro Chino, and Daiji Kambe. Conducted experiments: Shunsuke Kamigaso, Yoshihiro Konno, Yuichi Tokumaru, Hirohiko Hikichi, and Daiji Kambe. Performed data analysis: Shunsuke Kamigaso, Yoshihiro Konno, Haruyuki Mori, Hironori Yamasaki, and Yoko Mano. Wrote or contributed to the writing of the manuscript: Shunsuke Kamigaso, Yoshihiro Konno, Hirohiko Hikichi, Yoko Mano, Yukihiro Chino, Daiji Kambe, Kenji Hachiuma, and Akiko Mizuno‐Yasuhira.

## Funding

This work was supported by Taisho Pharmaceutical Co. Ltd.

## Disclosure

All authors are employees of Taisho Pharmaceutical Co. Ltd.

## Conflicts of Interest

All authors are employees of Taisho Pharmaceutical Co. Ltd.

## Data Availability

Research data are not shared.

## References

[1] S. Schutte‐Rodin , L. Broch , D. Buysse , C. Dorsey , and M. Sateia , “Clinical Guideline for the Evaluation and Management of Chronic Insomnia in Adults,” Journal of Clinical Sleep Medicine 4 (2008): 487–504. 18853708 PMC2576317

[2] V. Scharner , L. Hasieber , A. Sönnichsen , and E. Mann , “Efficacy and Safety of Z‐Substances in the Management of Insomnia in Older Adults: A Systematic Review for the Development of Recommendations to Reduce Potentially Inappropriate Prescribing,” BMC Geriatrics 22 (2022): 87. 35100976 10.1186/s12877-022-02757-6 PMC9887772

[3] T. Sakurai , A. Amemiya , M. Ishii , et al., “Orexins and Orexin Receptors: A Family of Hypothalamic Neuropeptides and G Protein‐Coupled Receptors That Regulate Feeding Behavior,” Cell 92 (1998): 573–585. 9491897 10.1016/s0092-8674(00)80949-6

[4] L. de Lecea , T. S. Kilduff , C. Peyron , et al., “The Hypocretins: Hypothalamus‐Specific Peptides With Neuroexcitatory Activity,” Proceedings National Academy of Sciences of the United States of America 95 (1998): 322–327. 10.1073/pnas.95.1.322 PMC18213 9419374

[5] C. Boss and C. Roch , “Recent Trends in Orexin Research–2010 to 2015,” Bioorganic & Medicinal Chemistry Letters 25 (2015): 2875–2887. 26045032 10.1016/j.bmcl.2015.05.012

[6] T. Sakurai , “The Neural Circuit of Orexin (Hypocretin): Maintaining Sleep and Wakefulness,” Nature Reviews. Neuroscience 8 (2007): 171–181. 17299454 10.1038/nrn2092

[7] C. J. Winrow and J. J. Renger , “Discovery and Development of Orexin Receptor Antagonists as Therapeutics for Insomnia,” British Journal of Pharmacology 171 (2014): 283–293. 23731216 10.1111/bph.12261 PMC3904252

[8] C. Penyron , J. Faraco , W. Rogers , et al., “A Mutation in a Case of Early Onset Narcolepsy and a Generalized Absence of Hypocretin Peptides in Human Narcoleptic Brains,” Nature Medicine 6 (2000): 991–997. 10.1038/79690 10973318

[9] T. C. Thannickal , R. Y. Moore , R. Nienhuis , et al., “Reduced Number of Hypocretin Neurons in Human Narcolepsy,” Neuron 27 (2000): 469–474. 11055430 10.1016/s0896-6273(00)00058-1 PMC8760623

[10] Y. Han , K. Yuan , Y. Zheng , and L. Lu , “Orexin Receptor Antagonists as Emerging Treatments for Psychiatric Disorders,” Neuroscience Bulletin 36 (2020): 432–448. 31782044 10.1007/s12264-019-00447-9 PMC7142186

[11] BELSOMRA (Suvorexant) [Package Insert] (Merck & Co. Inc., 2021), https://www.accessdata.fda.gov/scripts/cder/daf/index.cfm?event=overview.process&ApplNo=204569.

[12] DAYVIGO (Lemborexant) [Package Insert] (Eisai Inc, 2023), https://www.accessdata.fda.gov/scripts/cder/daf/index.cfm?event=overview.process&ApplNo=212028.

[13] QUVIVIQ (Daridorexant) [Package Insert] (Idorsia Pharmaceuticals US Inc, 2022), https://www.accessdata.fda.gov/scripts/cder/daf/index.cfm?event=overview.process&ApplNo=214985.

[14] T. Citrome , “Suvorexant for Insomnia: A Systematic Review of the Efficacy and Safety Profile for This Newly Approved Hypnotic—What Is the Number Needed to Treat, Number Needed to Harm and Likelihood to Be Helped or Harmed?,” International Journal of Clinical Practice 68 (2014): 1429–1441. 25231363 10.1111/ijcp.12568

[15] A. Vermeeren , H. Sun , E. F. P. M. Vuurman , et al., “On‐The‐Road Driving Performance the Morning After Bedtime Use of Suvorexant 20 and 40 mg: A Study in Non‐Elderly Healthy Volunteers,” Sleep 38 (2015): 1803–1813. 26039969 10.5665/sleep.5168 PMC4813357

[16] A. Futamura , R. Suzuki , Y. Tamura , et al., “Discovery of ORN0829, a Potent Dual Orexin 1/2 Receptor Antagonist for the Treatment of Insomnia,” Bioorganic & Medicinal Chemistry 28 (2020): 115489. 32482533 10.1016/j.bmc.2020.115489

[17] D. Kambe , S. Hasegawa , Y. Imadera , et al., “Pharmacokinetics, Pharmacodynamics, and Safety Profile of the Dual Orexin Receptor Antagonist Vornorexant/TS‐142 in Healthy Japanese Participants Following Single/Multiple Dosing: Randomized, Double‐Blind, Placebo‐Controlled Phase‐1 Studies,” Basic & Clinical Pharmacology & Toxicology 133 (2023): 576–591. 37563858 10.1111/bcpt.13930

[18] M. Uchiyama , D. Kambe , S. Hasegawa , et al., “Efficacy and Safety of Vornorexant in Japanese Patients With Insomnia: A Placebo‐Controlled Phase 3 Pivotal Study,” Sleep (2025): zsaf291. 41001841 10.1093/sleep/zsaf291 PMC13017578

[19] A. L. Gotter , C. J. Winrow , J. Brunner , et al., “The Duration of Sleep Promoting Efficacy by Dual Orexin Receptor Antagonists Is Dependent Upon Receptor Occupancy Threshold,” BMC Neuroscience 14 (2013): 90. 23981345 10.1186/1471-2202-14-90 PMC3765993

[20] A. Treiber , R. de Kanter , C. Roch , et al., “The Use of Physiology‐Based Pharmacokinetic and Pharmacodynamic Modeling in the Discovery of the Dual Orexin Receptor Antagonist ACT‐541468,” Journal of Pharmacology and Experimental Therapeutics 362 (2017): 489–503. 28663311 10.1124/jpet.117.241596

[21] P. Bonaventure , J. Shelton , S. Yun , et al., “Characterization of JNJ‐42847922, a Selective Orexin‐2 Receptor Antagonist, as a Clinical Candidate for the Treatment of Insomnia,” Journal of Pharmacology and Experimental Therapeutics 354 (2015): 471–482. 26177655 10.1124/jpet.115.225466

[22] A. L. Gotter , M. S. Forman , C. M. Harrell , et al., “Orexin 2 Receptor Antagonism Is Sufficient to Promote NREM and REM Sleep From Mouse to Man,” Scientific Reports 6 (2016): 27147. 27256922 10.1038/srep27147 PMC4891657

[23] H. Hikichi , Y. Tokumaru , A. Taruta , et al., “Preclinical Pharmacological Profiles of Vornorexant, a Novel Potent and Selective Dual Orexin Receptor Antagonist,” Journal of Pharmacology and Experimental Therapeutics 392 (2025): 103624. 40570549 10.1016/j.jpet.2025.103624

[24] Y. Konno , S. Kamigaso , H. Toki , et al., “Preclinical Metabolism and the Disposition of Vornorexant/TS‐142, a Novel Dual Orexin 1/2 Receptor Antagonist for the Treatment of Insomnia,” Pharmacology Research & Perspectives 12 (2024): e1183. 38491717 10.1002/prp2.1183 PMC10943176

[25] M. Uchiyama , D. Kambe , Y. Imadera , et al., “Effects of TS‐142, a Novel Dual Orexin Receptor Antagonist, on Sleep in Patients With Insomnia: A Randomized, Double‐Blind, Placebo‐Controlled Phase 2 Study,” Psychopharmacology (Berlin) 239 (2022): 2143–2154. 35296912 10.1007/s00213-022-06089-6 PMC9205809

[26] X. Shen , Y. Wu , D. Zhang , et al., “Nighttime Sleep Duration, 24‐Hour Sleep Duration and Risk of All‐Cause Mortality Among Adults: A Meta‐Analysis of Prospective Cohort Studies,” Scientific Reports 6 (2016): 21480. 26900147 10.1038/srep21480 PMC4761879

[27] J. Yin , X. Jin , Z. Shan , et al., “Relationship of Sleep Duration With All‐Cause Mortality and Cardiovascular Events: A Systematic Review and Dose‐Response Meta‐Analysis of Prospective Cohort Studies,” Journal of the American Heart Association 6 (2017): e005947. 28889101 10.1161/JAHA.117.005947 PMC5634263

[28] A. A. da Silva , R. G. de Mello , C. W. Schann , et al., “Sleep Duration and Mortality in the Elderly: A Systematic Review With Meta‐Analysis,” BMJ Open 6 (2016): e008119. 10.1136/bmjopen-2015-008119 PMC4762152 26888725

[29] M. Mieda , E. Hasegawa , Y. Y. Kisanuki , C. M. Sinton , M. Yanagisawa , and T. Sakurai , “Differential Roles of Orexin Receptor‐1 and ‐2 in the Regulation of Non‐REM and REM Sleep,” Journal of Neuroscience 31 (2011): 6518–6526. 21525292 10.1523/JNEUROSCI.6506-10.2011 PMC3732784

[30] M. A. Steiner , J. Gatfield , C. Brisbare‐Roch , et al., “Discovery and Characterization of ACT‐335827, an Orally Available, Brain Penetrant Orexin Receptor Type 1 Selective Antagonist,” ChemMedChem 8, no. 6 (2013): 898–903. 23589487 10.1002/cmdc.201300003

[31] P. Bonaventure , C. Dugovic , B. Shireman , et al., “Evaluation of JNJ‐54717793 a Novel Brain Penetrant Selective Orexin 1 Receptor Antagonist in Two Rat Models of Panic Attack Provocation,” Frontiers in Pharmacology 8 (2017): 357. 28649201 10.3389/fphar.2017.00357 PMC5465257

[32] C. Dugovic , J. E. Shelton , L. E. Aluisio , et al., “Blockade of Orexin‐1 Receptors Attenuates Orexin‐2 Receptor Antagonism‐Induced Sleep Promotion in the Rat,” Journal of Pharmacology and Experimental Therapeutics 330 (2009): 142–151. 19363060 10.1124/jpet.109.152009

[33] G. M. Mang , T. Dürst , H. Bürki , et al., “The Dual Orexin Receptor Antagonist Almorexant Induces Sleep and Decreases Orexin‐Induced Locomotion by Blocking Orexin 2 Receptors,” Sleep 35 (2012): 1625–1635. 23204605 10.5665/sleep.2232 PMC3490355

[34] D. Hoyer , T. Dürst , M. Fendt , et al., “Distinct Effects of IPSU and Suvorexant on Mouse Sleep Architecture,” Frontiers in Neuroscience 7 (2013): 235. 24368893 10.3389/fnins.2013.00235 PMC3857892

